# Using Structural Information to Change the Phosphotransfer Specificity of a Two-Component Chemotaxis Signalling Complex

**DOI:** 10.1371/journal.pbio.1000306

**Published:** 2010-02-09

**Authors:** Christian H. Bell, Steven L. Porter, Annabel Strawson, David I. Stuart, Judith P. Armitage

**Affiliations:** 1 Oxford Centre for Integrative Systems Biology, Department of Biochemistry, University of Oxford, Oxford, United Kingdom; 2 Division of Structural Biology, University of Oxford, Oxford, United Kingdom; Brandeis University, United States of America

## Abstract

Two-component signal transduction pathways comprising histidine protein kinases (HPKs) and their response regulators (RRs) are widely used to control bacterial responses to environmental challenges. Some bacteria have over 150 different two-component pathways, and the specificity of the phosphotransfer reactions within these systems is tightly controlled to prevent unwanted crosstalk. One of the best understood two-component signalling pathways is the chemotaxis pathway. Here, we present the 1.40 Å crystal structure of the histidine-containing phosphotransfer domain of the chemotaxis HPK, CheA_3_, in complex with its cognate RR, CheY_6_. A methionine finger on CheY_6_ that nestles in a hydrophobic pocket in CheA_3_ was shown to be important for the interaction and was found to only occur in the cognate RRs of CheA_3_, CheY_6_, and CheB_2_. Site-directed mutagenesis of this methionine in combination with two adjacent residues abolished binding, as shown by surface plasmon resonance studies, and phosphotransfer from CheA_3_-P to CheY_6_. Introduction of this methionine and an adjacent alanine residue into a range of noncognate CheYs, dramatically changed their specificity, allowing protein interaction and rapid phosphotransfer from CheA_3_-P. The structure presented here has allowed us to identify specificity determinants for the CheA–CheY interaction and subsequently to successfully reengineer phosphotransfer signalling. In summary, our results provide valuable insight into how cells mediate specificity in one of the most abundant signalling pathways in biology, two-component signal transduction.

## Abstract 2

Analysis of the crystal structure of a phosphotransfer complex from the *Rhodobacter sphaeroides* chemotaxis pathway allowed reengineering of molecular recognition in a two-component signalling system.

## Author Summary

The ability to respond to environmental stimuli is a universal feature of living cells. Evolution has created a vast array of signalling mechanisms that enable cells to react in many ways to extracellular changes. In bacteria, two-component signalling mechanisms, comprising a sensor protein kinase paired with its a cognate response regulator, are used widely to sense and respond to environmental changes. Some species of bacteria have over 150 different two-component pairs in a single cell, so the specificity between these pairs has to be tightly controlled to prevent “crossed wires” between signalling pathways. In this study, we have identified the determinants of this specificity in a two-component complex that controls the movement of *Rhodobacter sphaeroides* along a chemical gradient. By solving and analysing the crystal structure of this complex, we were able to pinpoint the amino acid residues that are crucially involved in formation of the complex. Knowledge of these crucial residues allowed us to convert noncognate response regulators into cognate response regulators simply by changing two amino acids. This reengineering of two-component signalling pathways paves the way for producing custom-designed circuits for applications in synthetic biology.

## Introduction

Bacteria, Archaea, and some eukaryotes use two-component signalling pathways to detect environmental conditions and bring about appropriate changes in cellular behaviour [1],[2]. Two-component pathways comprise sensor histidine kinases (HPK) and response regulators (RRs). Environmental stimuli control the rate at which the HPK autophosphorylates on a conserved histidine residue. Once phosphorylated, the HPK transfers the phosphoryl group to an aspartate residue within the receiver domain of the cognate RR. The phosphorylated RR (RR-P), often a transcriptional regulator, then effects a response appropriate to the original stimulus. Some bacteria have over 150 different HPK and RR pairs, and the specificity of the phosphorylation reactions between them needs to be tightly controlled to prevent HPKs from inappropriately phosphorylating and activating noncognate RRs. A number of mechanisms contribute to this specificity [3],[4], although the primary one is molecular recognition, in which a HPK shows a strong kinetic preference for its cognate RRs [5],[6]. Understanding the mechanisms involved in molecular recognition will not only allow prediction of interacting pairs, but potentially allow the rewiring of bacterial sensory pathways for use in synthetic biology.

Many two-component systems utilize an additional element, the Hpt (histidine-containing phosphotransfer) domain in phosphotransfer; these include multistep phosphorelays and chemotaxis signalling pathways. In multistep phosphorelays, the Hpt domain serves as an intermediate in the transfer of phosphoryl groups from the receiver domain phosphorylated by the HPK and the output RR. In contrast, the histidine within the Hpt domain is the initial site of phosphorylation in the chemotaxis HPK, CheA, and is phosphorylated using ATP as the phosphodonor [7]. Subsequently, the phosphoryl group is transferred from the histidine residue in the Hpt (P1) domain of CheA to an aspartate residue on either of two RRs, CheY or CheB [8]. In this study, we present the structure of the Hpt (P1) domain of CheA_3_ from *R. sphaeroides* in complex with its cognate RR CheY_6_, which, to our knowledge, is the first structure of a Hpt domain of a CheA protein in complex with its RR.

The chemotaxis pathway of *Escherichia coli* has been extensively characterized [9]–[12]. However, many bacteria have more complicated chemosensory pathways, employing multiple homologues of each of the chemosensory proteins [13]–[15]. *R. sphaeroides* has four CheA homologues and eight chemotaxis RR proteins (six CheYs and two CheBs) plus multiple homologues of the other *E. coli* chemotaxis proteins [13],[16]–[18]. Interestingly, the different CheAs show different phosphotransfer specificities for the CheY and CheB homologues [19]–[22]. CheA_3_ and CheA_4_ form a cluster with the soluble chemoreceptors in the cytoplasm, which is believed to sense the metabolic state of the cell [13],[23],[24]. CheA_3_ and CheA_4_ are unusual CheAs that lack some of the domains found in *E. coli* CheA [20],[25]. CheA_4_ has a P3 (dimerization) domain, a P4 (kinase) domain, and a P5 (regulatory) domain, whereas CheA_3_ has a P1 (Hpt) domain and a P5 (regulatory) domain separated by a 794-amino acid sequence that encodes a novel CheY-P phosphatase activity [26]. Neither CheA_3_ nor CheA_4_ is capable of autophosphorylation; instead, CheA_4_ phosphorylates CheA_3_ on H51 of the P1 (Hpt) domain. Once phosphorylated, CheA_3_-P serves as a specific phosphodonor for the chemotaxis RRs, CheY_1_, CheY_6_, and CheB_2_. However, it is unable to phosphorylate the other chemotaxis RRs, CheY_2_, CheY_3_, CheY_4_, CheY_5_, and CheB_1_ [20],[21],[27]. This phosphotransfer specificity must be determined by the interactions between the RRs and the P1 domain of CheA_3_ since the isolated P1 domain of CheA_3_ (CheA_3_P1) shows the same phosphotransfer specificity as full-length CheA_3_ [26].

In groundbreaking work, the Laub group used a mutual information bioinformatics approach to identify coevolving residues in HPKs and their cognate RRs [28]. They reasoned that many of the coevolving residues would be the specificity determinants for the phosphotransfer reaction and went on to show that it is possible to switch the RR substrate specificity of the HPK EnvZ by mutating these residues [28]. In this study, we use a structure-based approach to identify specificity determinants for the phosphotransfer reaction between CheA_3_P1-P and CheY_6_. By introducing these residues into the noncognate RRs, we have been able to change their kinase specificity, allowing them to be phosphorylated by CheA_3_P1-P.

## Results

### Structure of the CheA_3_P1.CheY_6_ Complex

In order to elucidate the molecular details of the interaction between CheA_3_ and CheY_6_, the complex structure of CheY_6_ and the unphosphorylated Hpt domain of CheA_3_ (residues 1–135) has been solved to 1.40 Å using Seleno single-wavelength anomalous dispersion (SAD) for phasing. Data collection and refinement statistics can be found in Table 1.

**Table 1:** Data collection and refinement statistics.

Data Collection and Refinement	Statistics	Subcategory	SeMet	Unphosphorylated	Phosphorylated
**Data collection**	Resolution (Å)		2.3 (2.38–2.30)^a^	1.40 (1.45–1.40)	2.80 (2.90–2.80)
	Space group		P2_1_	P2_1_	P1
	Cell dimension	*a*, *b*, *c* (Å)	43.8, 62.7, 49.1	43.7, 62.0, 48.9	33.2, 43.4, 48.7
		α, β, γ (°)	90, 101.1, 90	90, 101.3, 90	78.8, 86.6, 80.9
	Redundancy		9.5 (10.1)	6.3 (3.7)	1.8 (1.8)
	Completeness (%)		99.2 (99.0)	99.7 (98.9)	96.1 (94.5)
	Rsym (%)		8.9 (19.7)	5.3 (14.8)	12.0 (61.6)
	Avg I/σ		18.4 (12.9)	31.6 (9.8)	6.3 (1.2)
**Refinement**	Resolution			38–1.40	42–2.80
	No. reflections			50287	6232
	*R*_work_/*R*_free_			17.0/20.7	24.1^b^/28.4^b^
	No. atoms	Protein		2040	1743
		Water		272	—
	B-factors (Å^2^)	Protein		28.5	86.5^c^
		Water		38.9	—
	r.m.s. deviations	Bond lengths (Å)		0.010	0.008
		Bond angles (°)		1.220	0.950

a Numbers in parenthesis are for the highest resolution shell.

b Rxpct, as described elsewhere [43].

c Standard deviation of ±18 Å^2^.

**Figure 1:**
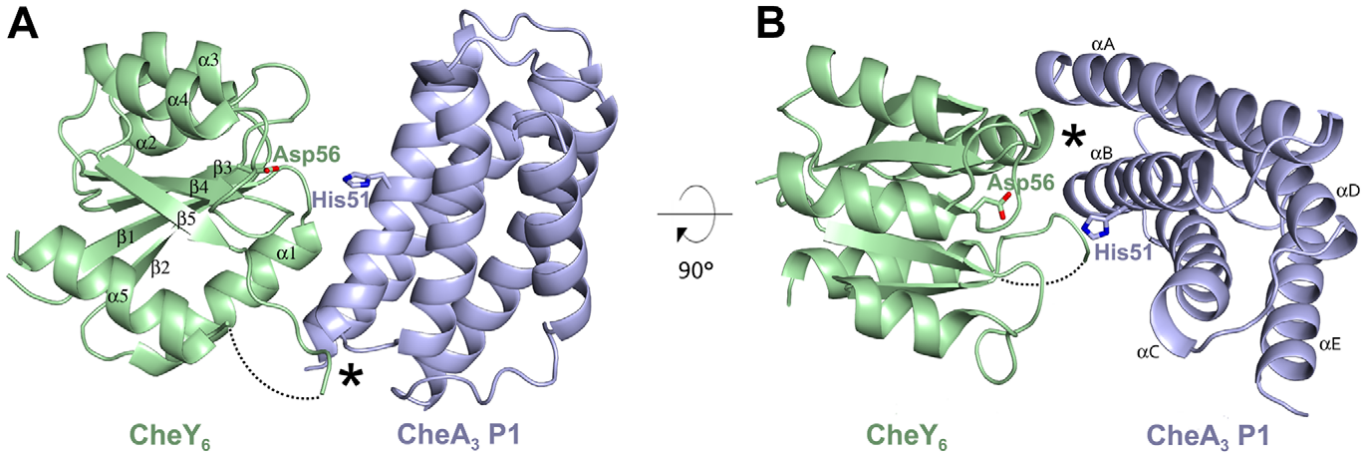
Structure of the CheA_3_P1.CheY_6_ complex. CheA_3_P1 is shown in light blue whereas CheY_6_ is in pale green. The phosphorylatable residue of CheA_3_P1, His51, and the phosphoacceptor on CheY_6_, Asp56, are shown in stick representation. Secondary structure elements are labelled in black. Residues 113–118 of CheY_6_ could not be traced and are depicted as a dotted line. (A) Side view onto the CheA_3_P1.CheY_6_ complex with the interaction between β5 and α5 in CheY_6_ and αB, and the following loop connecting αB and αC on CheA_3_P1, marked with an asterisk. (B) Top view showing the major site of interaction between the N-terminal end of α1 on CheY_6_ and αA/αB on CheA_3_P1 marked by an asterisk.

CheY_6_ has the typical (α/β)_5_ topology seen for *E. coli* CheY [29] and other structurally characterized RRs (Figure 1). Comparison with *E. coli* CheY reveals a high degree of structural conservation with a root mean square deviation (rmsd) of 1.4 Å over 115 Cα atoms (26% sequence identity). Although crystallized in the presence of Mn^2+^, there was no additional density in the CheY_6_ divalent cation binding site. However, the conformation of the metal coordinating residues Asp56, Asp9, and Asp10 is most similar to that found in the structure of Mg^2+^ bound CheY [30]. Only the backbone carbonyl of Glu58 is facing away rather than pointing towards the potential metal binding site (Figure S1). CheY_6_ has an elongated loop region connecting β5 and α5 (Figure 1). This loop comprises 13 residues (residues 107–119) in CheY_6_ compared to only three residues (109–111) in *E. coli* CheY. In the crystal, this loop is only partially ordered, suggesting that it is highly flexible. Residues 113–118 could not be traced and were omitted from the final model. The N-terminal region of this β5-α5 loop in combination with α1 of CheY_6_ form the vast majority of contacts to CheA_3_P1 (Figure 1).

CheA_3_P1 forms a four-helix bundle (αA–αD) with an additional C-terminal helix (αE) (Figure 1). Despite low sequence identity, it is structurally very similar to previously determined CheA P1 structures [31]–[33]. Comparison with CheA P1 from *Salmonella enterica* serovar Typhimurium [32] and *Thermotoga maritima* [33] gives an rmsd of 1.2 Å over 116 Cα atoms (21% sequence identity) and 1.5 Å over 101 Cα (18% sequence identity), respectively. The site of phosphorylation, His51 in CheA_3_P1, is located on αB, in close proximity to the active site of the RR (Figure 1) with the phosphoacceptor Asp56 on CheY_6_ being 7.5Å from His51 on CheA_3_P1. Helices αA and αB face the RR and together with the αB–αC loop form the interface with CheY_6_ (Figure 1).

### Structure of Phosphorylated CheA_3_P1 in Complex with CheY_6_(D56N, S83A)

To show that the conclusions drawn from the unphosphorylated structure are also valid for the physiologically relevant, phosphorylated complex, we solved the structure of phosphorylated CheA_3_P1 in complex with CheY_6_. Since the rapid rate of phosphotransfer between CheA_3_P1-P and CheY_6_ does not allow crystallization of the wild-type, phosphorylated complex, we formed a stable, complex by introducing two substitutions, D56N and S83A, in the active site of CheY_6_ (see Materials and Methods for details). These substitutions have previously been shown to abolish phosphotransfer from CheA_3_P1-P [19]. The structure was solved to 2.8 Å by molecular replacement using the unphosphorylated structure as model. Data collection and refinement statistics can be found in Table 1. The structure shows clear additional density adjacent to Nε2 of His51 of CheA_3_P1 in agreement with phosphorylation of this residue (Figure S2). Due to the moderate resolution of the analysis, residues 60–65, 85–97, and 111–121 on CheY_6_ could not be traced and were not included in the final model.

**Figure 2:**
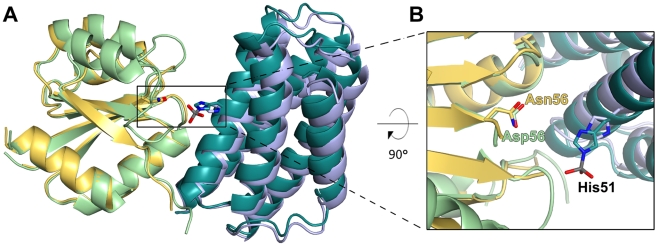
Superposition of the CheA_3_P1.CheY_6_ complex in the phosphorylated and unphosphorylated conformations. Structures were aligned onto the RR. Colour coding for the unphosphorylated conformation as in Figure 1; for the phosphorylated conformation, CheY_6_ is shown in yellow and CheA_3_ P1 in teal. The active site residues His51 (CheA_3_ P1) and Asp56/Asn56 (CheY_6_ unphosphorylated/phosphorylated complex conformation) are shown in stick representation. (A) Overview highlighting the 2.1 Å rigid body translation of CheA_3_ P1 towards CheY_6_. (B) Close-up view onto the active site showing the movement of His51 towards Asp56/Asn56.

Compared to the unphosphorylated complex, CheA_3_P1-P undergoes a rigid body translation of 2.1 Å relative to the RR (Figure 2A). This realignment of CheA_3_P1-P positions the phosphorylated His51 closer to the phosphoacceptor on CheY_6_, Asp56 (Figure 2B). The phosphorylated His51 is facing away from the active site of CheY_6_; however, a 180° flip of this side chain would put it in near linear geometry and place the phosphoryl group within 4.5 Å of Asp56 of CheY_6_ (Figure 2B). The orientation of the phosphorylated His51 seen in the crystal structure is likely caused by the lack of a divalent cation in the metal binding site of the RR leading to electrostatic repulsion from the CheY_6_ active site.

The difference in binding affinity for CheY_6_ between CheA_3_ and CheA_3_-P is not known. In *E. coli*, the difference in *K_d_* values for CheY between CheA and CheA-P is relatively small, with both values being in the low micromolar range [34]. Consistent with this, we find very little change in the total buried surface area between the phosphorylated (605 Å^2^) and unphosphorylated (530 Å^2^) structures of CheA_3_P1 complexed with CheY_6_. The only difference is a hydrogen bond formed between Glu58 of CheY_6_ and His51 of CheA_3_P1 in the unphosphorylated complex that is released in the phosphorylated complex. As this is not contributing to the interface of the physiologically important complex, it is therefore not discussed further. Because the interfaces in the phosphorylated and unphosphorylated complex are highly similar, the following discussion of the interactions between CheA_3_P1 and CheY_6_ are based on the high-resolution, unphosphorylated structure.

### Interactions between CheA_3_P1 and CheY_6_

The interface between CheA_3_P1 and CheY_6_ is dominated by hydrophobic interactions with only one hydrogen bond and no salt bridges formed between the two proteins. The buried surface area of the interface is small (530 Å^2^), indicative of a weak interaction, consistent with the transient nature of the complexes [35]. A continuous interface is formed by two sites of interaction complemented by a hydrogen bond formed between Ser83 on CheY_6_ and Arg58 on CheA_3_P1. One of these sites lies between the elongated loop region between β5 and α5 in CheY_6_ and αB and the following loop connecting αB and αC on CheA_3_P1 (Figure 1A). The other site lies between the N-terminal end of α1 on CheY_6_ and αA/αB on CheA_3_P1 (Figure 1B). The latter is the major binding site including ∼70% of the total buried surface area (358 Å^2^).

**Figure 3:**
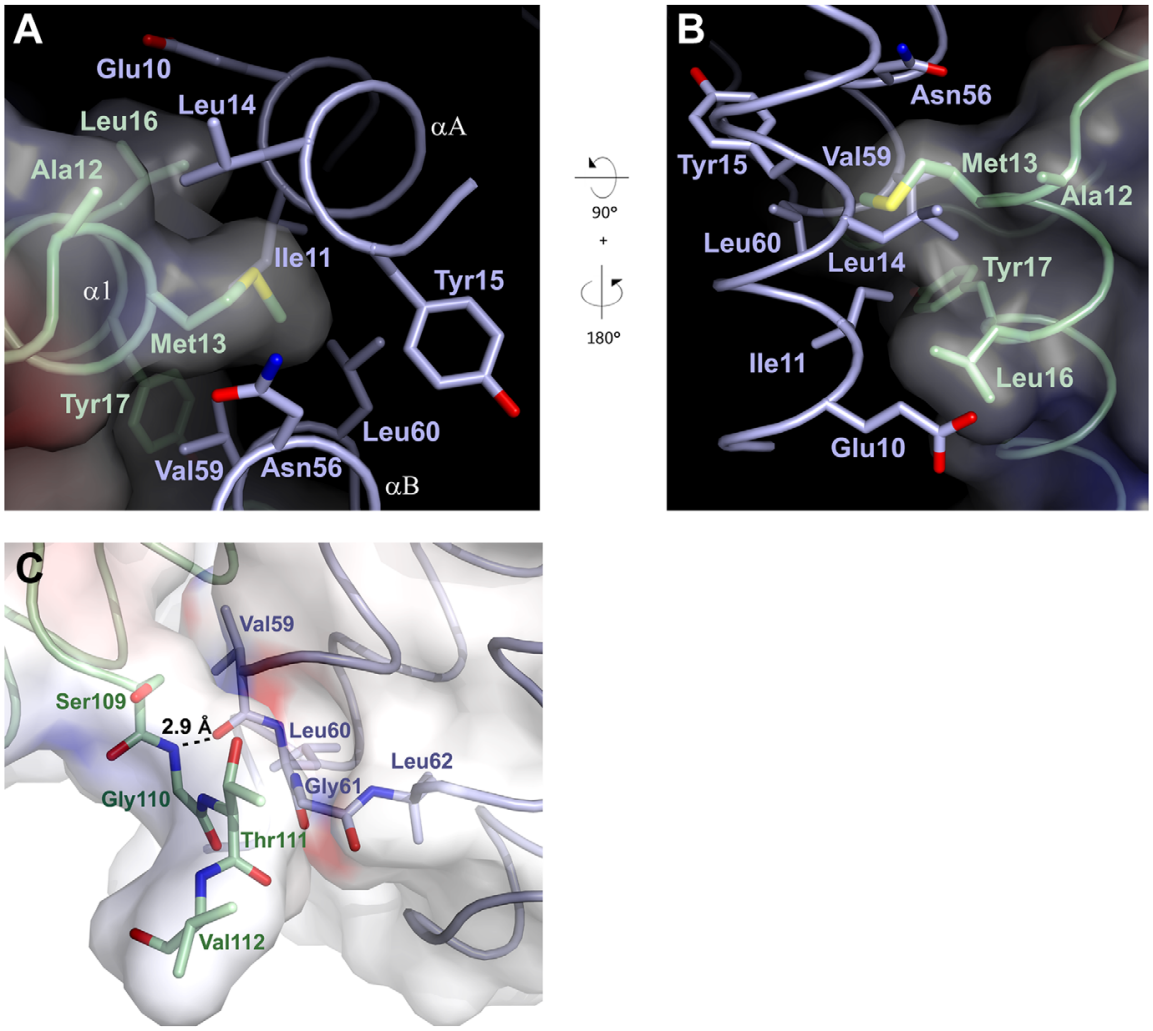
Close-up view of the two binding sites between CheA_3_P1 and CheY_6_. Colour coding as in Figure 1. Residues that are involved in the interaction are shown in stick representation. (A) Detailed view onto the major binding site between CheY_6_ and CheA_3_P1. Orientation as in Figure 1B, residues 19–53 of CheA_3_ P1 are omitted for clarity. (B) Solvent accessible surface of CheY_6_ coloured by electrostatic potential contoured at ±10 kT. Met13 fits snugly into the hydrophobic pocket on CheA_3_P1. (C) Close-up view onto the second binding site. Orientation as in Figure 1A. The solvent-accessible surface area is coloured by electrostatic potential contoured at ±10 kT. The hydrogen bond between Gly110 of CheY_6_ and Val59 of CheA_3_P1 is marked with a dotted line.

In this region, helices αA and αB of CheA_3_P1 form a hydrophobic pocket comprising Ile11, Leu14, and Tyr15 on αA, and Asn56, Val59, and Leu60 on αB (Figure 3A and 3B). This pocket is situated adjacent to α1 on CheY_6_ and in ideal position to accommodate Met13 on the N-terminal region of this helix. This Met finger is protruding from α1, and its side chain fits snugly into the hydrophobic pocket, with 99.7% of its accessible surface area being buried (Figure 3B). The hydrophobic interaction is complemented by Ala12, Leu16, and Tyr17 of α1 on CheY_6_, all facing the same side on CheA_3_P1 as Met13 (Figure 3A). Ala12 of CheY_6_ extends the hydrophobic interface by interaction with Leu14 of CheA_3_P1, whereas Leu16 of CheY_6_ interacts with Ile11 and the aliphatic part of Glu10 on αA of CheA_3_P1. Tyr17 extends the binding surface towards the β5-α5 loop by interacting with Val59 at the C-terminus of αB. The elongated loop between β5 and α5 of CheY_6_ forms van der Waals interactions with Ser109, Gly110, and Thr111 of CheY_6_ contacting Ile59, Gly61, and Ser63 on αB and the following loop region on CheA_3_P1 (Figure 3C). A main chain hydrogen bond is formed between Gly110 and Val59.

Of these interactions, the Met13 finger has the largest contribution towards the binding interface on CheY_6_, accounting for almost a third (156 Å^2^) of the total buried surface area. Together with Ala12, Leu16, and Tyr17, it is accounting for over 60% (321 Å^2^) of the interface. On CheA_3_P1, the residues involved in the interaction with CheY_6_ are slightly less clustered and spatially farther apart. Yet the three residues with the biggest contribution towards the total buried surface area, Val59 (103 Å^2^, 20% of total buried surface area), Leu14 (64 Å^2^, 12%), and Ile11 (48 Å^2^, 9%), together account for almost half of the overall binding surface.

**Figure 4:**
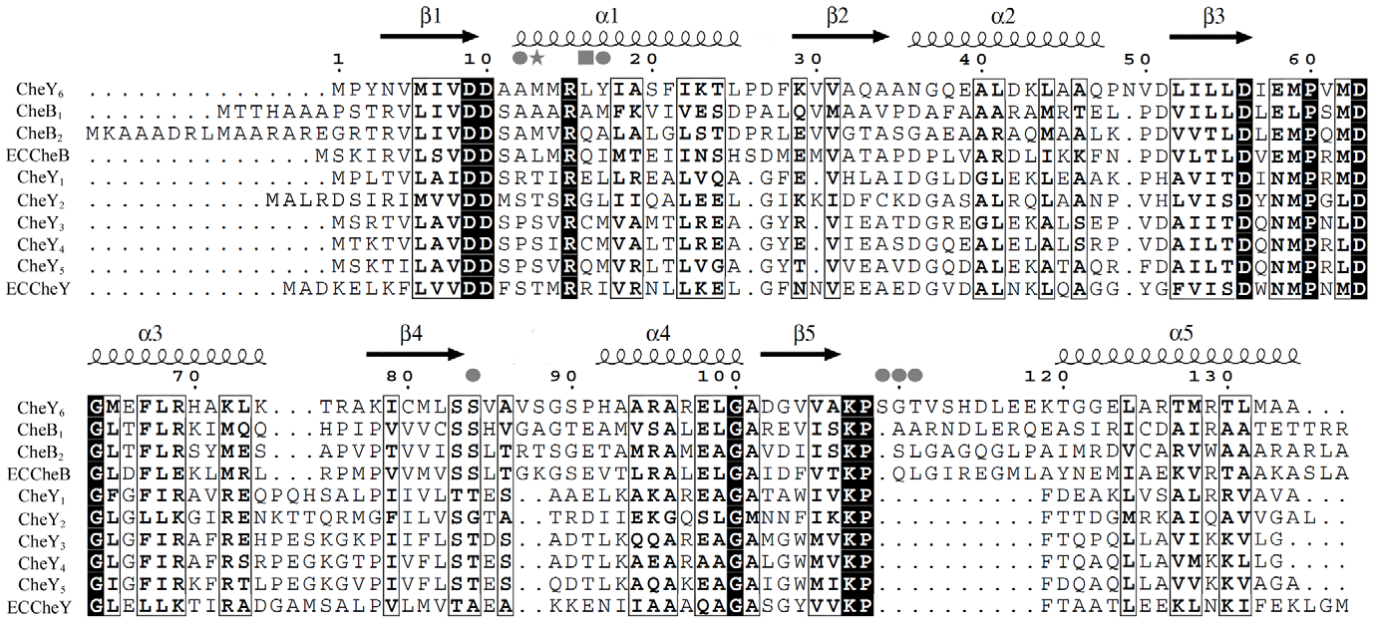
Structure-guided sequence alignment of the chemotaxis RRs from *E. coli* and *R. sphaeroides*. Alignment is based on the structures of *E. coli* CheY (PDB code: 3CHY), *E. coli* CheB (1A2O), *R. sphaeroides* CheY_3_ (C. H. Bell, unpublished data), and *R. sphaeroides* CheY_6_. Secondary structure is shown for CheY_6_. Residues involved in binding of CheY_6_ to CheA_3_P1 are marked with a star (contributes >30% to total buried surface area), square (>15%), or circle (>5%).

This analysis shows that only a small number of residues are necessary to form the majority of interactions within the complex. On CheY_6_, these are all located on α1. Within this helix, Met13 has the most crucial role in the hydrophobic interaction. A sequence alignment of all identified RRs in *R. sphaeroides* shows that only CheY_6_ and CheB_2_ have a Met residue at position 13, whereas all others either have a Ser, Thr, or Ala at this position (Figure 4). As CheA_3_-P serves as a phosphodonor for both CheY_6_ and CheB_2_ this suggests that Met13 has an important role in determining the specificity for binding to CheA_3_P1.

### Substitution of M13 in CheY_6_ Reduces Interaction and the Rate of Phosphotransfer from CheA_3_P1-P

Residues 11–17 of CheY_6_ collectively account for ∼67% of the buried surface area of CheY_6_ in the CheA_3_P1-CheY_6_ complex, with M13, L16, and Y17 each contributing ∼30%, ∼16%, and ∼8%, respectively. To confirm that this surface of CheY_6_ is involved in the interactions with CheA_3_P1-P that lead to phosphotransfer in solution, we substituted residues at this surface to mimic those found in the noncognate RRs, CheY_3_ and CheY_4_. M13 was changed to S, and Y17 was changed to M as found in both CheY_3_ and CheY_4_. CheY_3_ and CheY_4_ both have C at position 16; however, it has previously been shown that CheY_3_(C16S) and CheY_4_(C16S) behave indistinguishably from CheY_3_ and CheY_4_ in phosphotransfer assays (S. L. Porter, unpublished data), and therefore, to avoid any potential problems from disulphide bond formation during the purification, we changed L16 to S rather than C. Two mutant proteins were produced, CheY_6_(M13S) and CheY_6_(M13S,L16S,Y17M).

**Figure 5:**
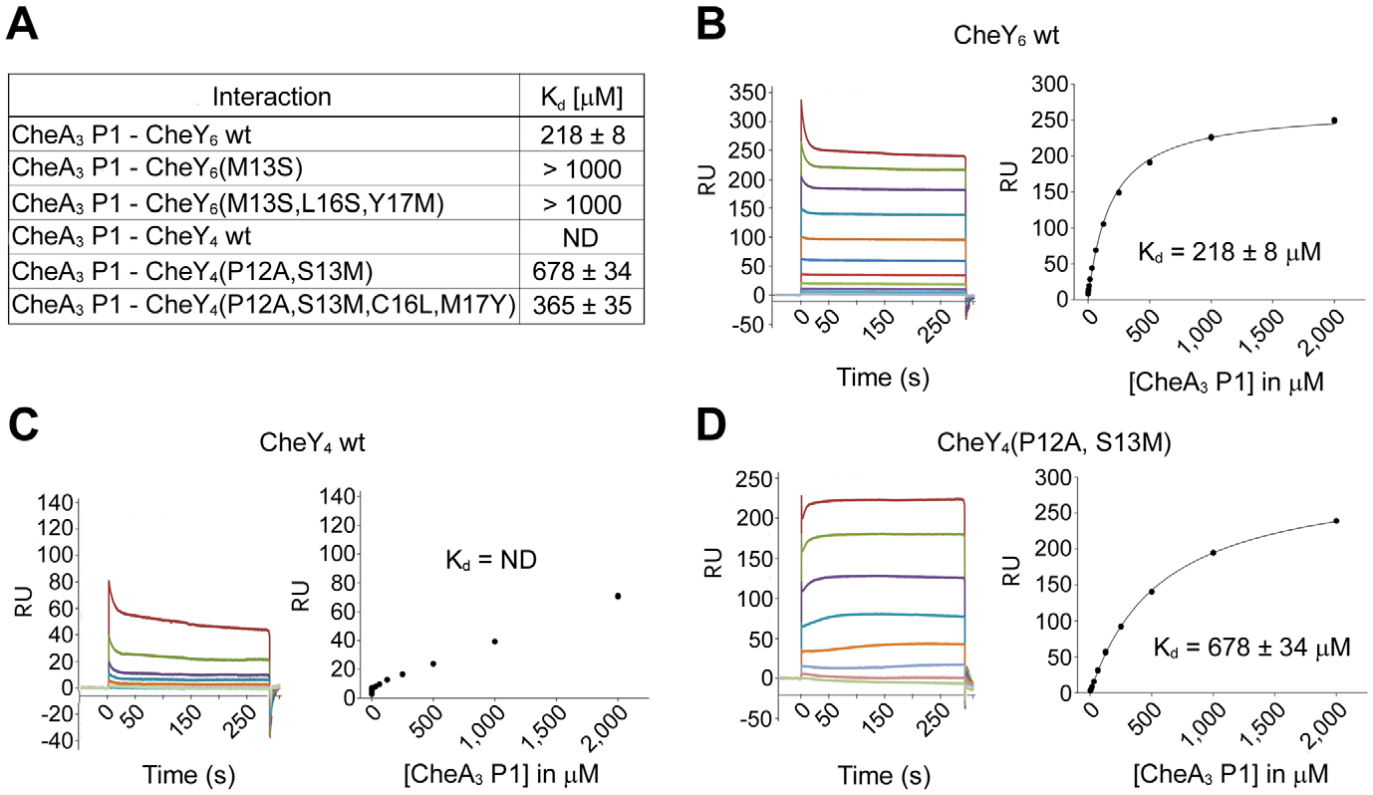
Binding of CheA_3_P1 to the response regulators. (A) Table of binding constants (*K_d_*) measured by SPR between CheA_3_P1 and CheY_4_, CheY_6_ and their mutant versions. Data are expressed as mean ± standard error of the mean (s.e.m.). ND, not determinable. (B–D) Binding of CheA_3_P1 to CheY_6_ wild type (wt), CheY_4_ wt, and CheY_4_(P12A,S13M). Left, representative sets of experimental sensorgrams from typical equilibrium-based binding experiments, with reference subtraction. Different concentrations of CheA_3_P1 were injected over surfaces coupled with the respective RR. For all injections, the experimental traces reached equilibrium and returned to baseline after the injection. Right, plot of the equilibrium binding response (response units [RU]) against CheA_3_P1 concentration ranging from 120 nM to 2 mM. Within one experiment, each concentration was measured twice. All experiments were performed in duplicate. Best-fit binding curves corresponding with a 1∶1 binding model are shown as lines.

Surface plasmon resonance (SPR) assays showed that wild-type CheY_6_ binds to CheA_3_P1 with an affinity of 218 µM (Figure 5). This weak interaction is in agreement with the transient nature of the complex and the small buried surface area between the two proteins as observed in the crystal structure. Both the single-mutant protein, CheY_6_(M13S), and the triple-mutant protein CheY_6_(M13S,L16S,Y17M) showed a remarkable decrease in affinity, with a *K_d_* of more than 1 mM (Figure 5 and Figure S3).

**Figure 6:**
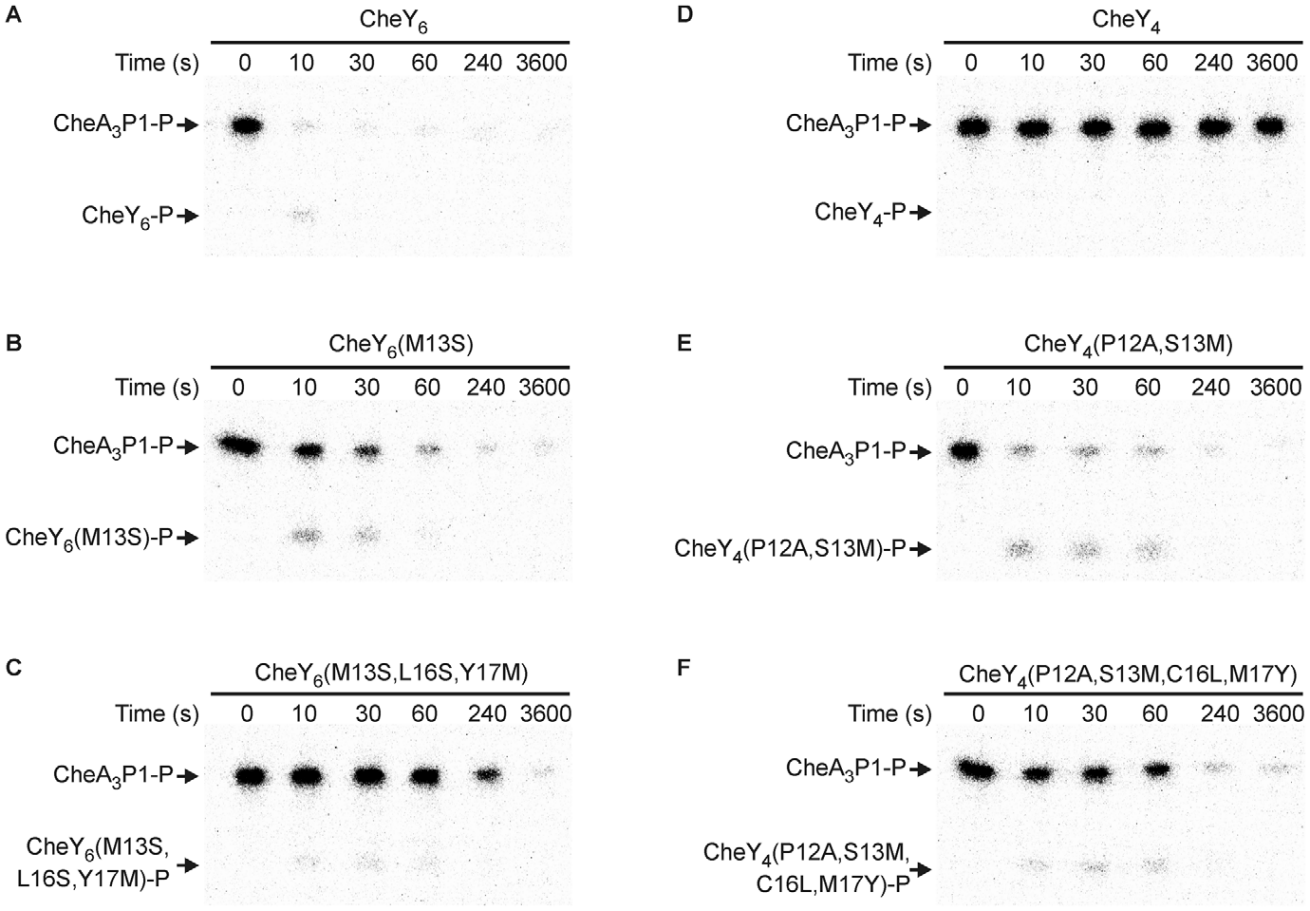
Changing the phosphotransfer specificity of CheY_6_ and CheY_4_. Phosphorimages of SDS-PAGE gels measuring phosphotransfer from CheA_3_P1-P to (A) CheY_6_, (B) CheY_6_(M13S), (C) CheY_6_(M13S,L16S,Y17M), (D) CheY_4_, (E) CheY_4_(P12A,S13M), and (F) CheY_4_(P12A,S13M,C16L,M17Y). CheY (5 µM) was added to 1 µM CheA_3_P1-^32^P. Ten-microlitre reaction samples were taken at the time points indicated and quenched in 20 µl of 1.5× SDS/EDTA loading dye. The quenched samples were analyzed by SDS-PAGE and detected by phosphorimaging.

Consistent with the SPR binding assays, the rate of phosphotransfer from CheA_3_P1-P to each of the two mutant CheY_6_ proteins was much slower than to wild-type CheY_6_ (Figure 6A–6C), with phosphotransfer to the triple-mutant protein CheY_6_(M13S,L16S,Y17M) being slowest. These results underline the conclusions drawn from the structural characterization of the complex and stress the importance of the Met finger at position 13 for binding of CheY_6_ to CheA_3_P1. In addition, the hydrophobic interaction mediated by Leu16 and Tyr17 also adds significantly to recognition of CheY_6_ by CheA_3_P1.

### Reengineering CheY_4_ into a Cognate RR of CheA_3_P1-P

The sequence of CheY_4_ is 36% identical to CheY_6_; however, unlike CheY_6_, CheY_4_ is not a cognate RR of CheA_3_P1-P (Figure 6D). Consistent with this, SPR assays failed to detect a significant interaction between CheA_3_P1 and CheY_4_ (Figure 5). In an attempt to alter the phosphotransfer specificity of CheY_4_ to allow phosphotransfer from CheA_3_P1-P, we substituted the CheY_4_ residues corresponding to the positions shown to interact in the CheA_3_P1-CheY_6_ structure, so that they matched CheY_6_. Two mutant proteins were produced: CheY_4_(P12A,S13M) and CheY_4_(P12A,S13M,C16L,M17Y). Ala12 was included in both mutant proteins since CheY_4_ has a Pro at position 12 that might influence the orientation of α1 and thus interfere with the proper positioning of Met13 for interaction with the hydrophobic pocket. SPR assays showed that both mutant proteins bind CheA_3_P1 (Figure 5 and Figure S3), and in phosphotransfer assays, both mutant proteins were phosphorylated by CheA_3_P1-P (Figure 6E and 6F). Phosphotransfer was fastest from CheA_3_P1-P to CheY_4_(P12A,S13M), with most of the initial CheA_3_P1-P dephosphorylated within 10 s (Figure 6E).

Interestingly, although the rate of phosphotransfer from CheA_3_P1-P to CheY_4_(P12A,S13M) was faster than to CheY_4_(P12A,S13M,C16L,M17Y), the SPR results show that CheA_3_P1 interacts slightly more strongly with CheY_4_(P12A,S13M,C16L,M17Y) than with CheY_4_(P12A,S13M) (Figure 5 and Figure S3). This apparent discrepancy could be explained by the alignment of the phosphorylated histidine and the phosphorylatable aspartate in the CheY_4_(P12A,S13M,C16L,M17Y).CheA_3_P1-P complex, which might be slightly less optimal for catalysis than in the CheY_4_(P12A,S13M).CheA_3_P1-P complex. Nevertheless, both methods show that mutating CheY_4_ so that it resembles CheY_6_ at the contact sites with CheA_3_P1 enhances both binding affinity and phosphotransfer rate. These results demonstrate that substitution of just two residues is sufficient to change the phosphotransfer specificity of CheY_4_.

### Reengineering Other CheYs

**Figure 7:**
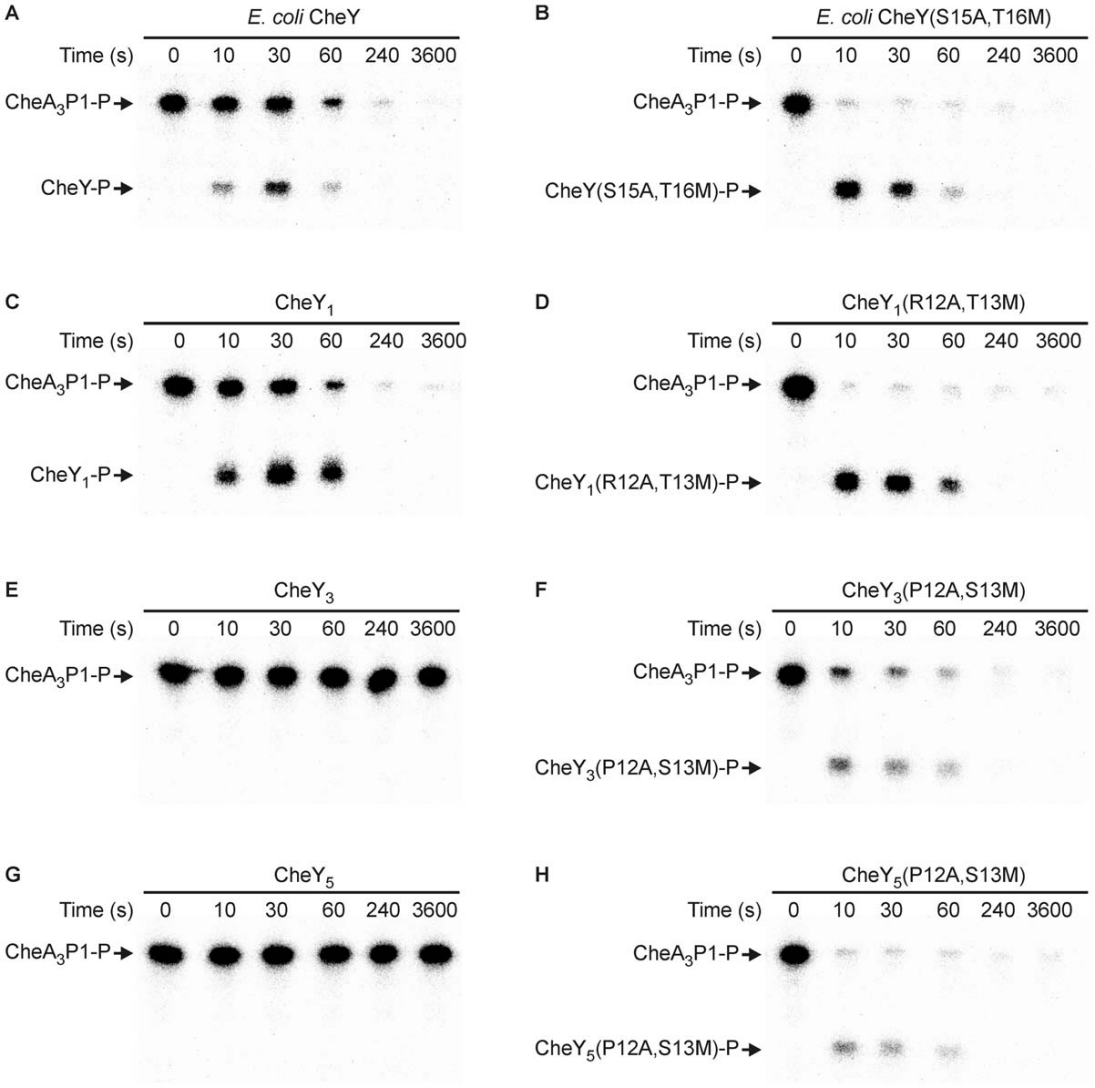
Changing the phosphotransfer specificity of other CheYs by introduction of A12 and M13. Phosphorimages of SDS-PAGE gels measuring phosphotransfer from CheA_3_P1-P to (A) *E. coli* CheY, (B) *E. coli* CheY(S15A,T16M), (C) CheY_1_, (D) CheY_1_(R12A,T13M), (E) CheY_3_, (F) CheY_3_(P12A,S13M), (G) CheY_5_, and (H) CheY_5_(P12A,S13M). CheY (5 µM) was added to 1 µM CheA_3_P1-^32^P. Ten-microlitre reaction samples were taken at the time points indicated and quenched in 20 µl of 1.5× SDS/EDTA loading dye. The quenched samples were analyzed by SDS-PAGE and detected by phosphorimaging.

Having successfully reengineered the phosphotransfer specificity of CheY_4_ by introducing A12 and M13, we used the same approach to change the specificity of CheY_1_, CheY_3_, CheY_5_, and *E. coli* CheY (Figure 7). These proteins share between 30% and 33% sequence identity with CheY_6_. In all cases, the mutant proteins containing the alanine and methionine substitutions were phosphorylated more rapidly by CheA_3_P1-P than were their wild-type counterparts. Although CheY_1_ and *E. coli* CheY both lack a methionine residue at the position corresponding to M13 of CheY_6_, they are both phosphorylated by CheA_3_P1-P (Figure 7A and 7C); however, phosphotransfer to their corresponding alanine and methionine substitution mutant proteins proceeded even faster, with almost complete dephosphorylation of CheA_3_P1-P within 10 s (Figure 7B and 7D). Similar to CheY_4_, wild-type CheY_3_ and CheY_5_ were not phosphorylated by CheA_3_P1-P (Figure 7E and 7G), and the effect of the alanine and methionine substitutions was to allow rapid phosphotransfer from CheA_3_P1-P (Figure 7F and 7H). These results demonstrate that the RR residues at the positions equivalent to A12 and M13 of CheY_6_ play a major role in determining phosphotransfer specificity.

## Discussion

Two-component signalling systems depend on a high level of selectivity between histidine kinases and their cognate RRs to prevent cross-talk between different pathways. Here, we present structural and functional data that elucidate how this specificity is mediated on a molecular level. We identified the key residues for molecular recognition and thereby were able to reengineer phosphotransfer specificity. Considering the high level of structural homology within the receiver and Hpt domain families, the presented structure together with complementary information, e.g., analysis of amino acid covariation [28],[36],[37], is likely to facilitate the rational reengineering of CheA-like histidine kinase–RR pairs for use, for example, in synthetic signalling circuits.

### Conserved Features of the CheA_3_P1.CheY_6_ Complex

The structure of CheA_3_P1 in complex with CheY_6_ is, to our knowledge, the first structure of a Hpt domain of a CheA protein in complex with a RR. To date, there are only two other structures of a Hpt domain in complex with a RR, namely Spo0B/Spo0F [38],[39] from *Bacillus subtilis* and YPD1/SLN1 [40],[41] from *Saccharomyces cerevisiae*. Although the overall structure of the RR is well conserved in all three complexes, there are remarkable differences in the Hpt domains. Spo0B exists as a dimer in which the two α-helices of the helical hairpin domain of each protomer associate to form a four-helix bundle. In addition, Spo0B has a C-terminal domain with an α/β fold, which is involved in binding of the RR. YPD1 is a monomeric Hpt protein consisting of a four-helix bundle with an additional short helix at the N-terminus. This N-terminal helix is involved in RR binding and complements the interface formed by helices αB and αC. When the three complexes were superimposed with respect to the receiver domains, CheA_3_P1 aligned well with YPD1 but only poorly with Spo0B (Figure S4).

### Phosphotransfer Specificity Determinants

CheA_3_P1.CheY_6_ shows the smallest binding interface amongst the three complexes solved so far, with 530 Å^2^ compared to 953 Å^2^ in YPD1/SLN1 and 1,200 Å^2^ in Spo0F/Spo0B. The interface is smaller since CheA_3_P1 has neither the C-terminal α/β fold domain seen in Spo0B nor the additional N-terminal helix seen in YPD1. Despite this small interface, CheA_3_P1 shows binding and efficient phosphotransfer to CheY_6_. It was previously proposed that the P2 domain of CheA-like proteins might be necessary for binding of the RR to CheA by increasing the binding interface [40]. However, CheA_3_ does not have a P2 domain, and the experimental data presented here and elsewhere [26] show that the P1 domain alone is sufficient for binding of and specific phosphotransfer to the cognate RR. Moreover, the structural and mutational analysis suggests that their interaction is mediated by very distinct residue clusters on the RR and the Hpt domain, the former being located on the N-terminal region of α1 and the loop region between β5 and α5, and the latter being located on the N- and C-terminal region of αA and αB, respectively, and the loop region connecting αB and αC. This is in good agreement with a recently published computational analysis of amino acid coevolution of cognate histidine kinase–RR pairs [28] and the binding site found in the YPD1/SLN1 [40],[41] or Spo0B/Spo0F [38],[39] complex.

### Reengineering Phosphotransfer Specificity

The specificity of protein–protein interactions is essential for most cellular processes. Despite the vast number of these interactions, our understanding of the molecular basis of their specificity is limited. The structure presented here has allowed us to identify specificity determinants for the CheA–CheY interaction. We successfully used this information to redesign noncognate RRs to allow them to be rapidly phosphorylated by CheA_3_P1-P (Figures 6 and 7). Whereas the Laub group have reengineered a HPK to specifically phosphorylate non-cognate RR substrates [28], we have now shown that it is possible to rationally reengineer a RR so that it can be phosphorylated by a noncognate HPK. The changing of phosphotransfer specificity described here represents, to our knowledge, the first example of the redesign of the intracellular part of the chemotaxis pathway and provides valuable insight into how cells mediate specificity in one of the most abundant signal transduction pathways, two-component signalling. The ability to reengineer phosphotransfer specificity coupled with recent work from the Bourret group [42], which has shown that RR autodephosphorylation rate can be manipulated, provide a platform for the future design of synthetic two-component circuits with customizable kinetics.

## Materials and Methods

### Protein Overexpression and Purification

CheA_3_P1 (residues 1–135 of CheA_3_, GenBank ID 3720125), CheY_6_ (GenBank ID 3720126), and CheY_4_ (GenBank ID 3722004) were cloned into the bacterial expression vector pQE80 (Qiagen), which includes an N-terminal His_6_-tag. Sequence verified plasmids were transformed into M15pREP4 cells (Qiagen) and cultivated in Terrific Broth to an absorbance at 600 nm (A_600_) of 0.8. Cultures were cooled to 20°C, induced with 0.25 mM IPTG, and then grown for ∼15 h before harvesting. The bacterial pellets were resuspended in 25 mM sodium phosphate (pH 8.0), 500 mM NaCl, 0.5 mM β-mercaptoethanol, and EDTA-free protease inhibitor cocktail (Roche). Cells were lysed using a Basic Z model cell disruptor (Constant Systems) and fractionated by centrifugation (45,000*g*, 4°C, 60 min). CheA_3_P1, CheY_6_, and CheY_4_ were purified from the supernatant by immobilized metal-affinity chromatography [19]–[21]. The samples were dialysed against standard buffer (30 mM Hepes [pH 7.5], 150 mM NaCl, 5 mM sodium acetate, 2 mM manganese chloride, 2 mM Tris(2-carboxyethyl)phosphine [TCEP]) and further purified by size-exclusion chromatography. Protein purity and concentration was measured as described [43]. Purified proteins were stored at −80°C. Seleno-methionine (SeMet)-labelled CheY_6_ was produced essentially as described [44]. The complex between CheY_6_ and CheA_3_P1 was formed by mixing equimolar amounts of both proteins. The sample was incubated for 30 min at room temperature (20°C), and the complex was purified by size-exclusion chromatography. CheA_3_P1 was phosphorylated using ATP and CheA_4_, and purified as described previously [26]. The final preparation of CheA_3_P1-P was free of ATP and CheA_4_. Phosphorylation was verified by mass spectrometry. Equimolar amounts of phosphorylated CheA_3_P1 and CheY_6_(D56N,S83A) were mixed and set up for crystallization.

### Site-Directed Mutagenesis

Mutations were introduced using the Quikchange Site-Directed Mutagenesis Kit (Stratagene).

### Crystallization and Data Collection

Crystal trials were set up with the purified CheA_3_P1.CheY_6_ complex at a concentration of 20 mg/ml. We set up nanoliter crystallization trials using a Cartesian Technologies robot (100 nl of protein solution plus 100 nl of reservoir solution) in 96-well Greiner plates [45], placed them in a TAP (The Automation Partnership) Homebase storage vault maintained at 295 K, and imaged them via a Veeco visualization system. Crystals of the phosphorylated, unphosphorylated and SeMet-labelled complex between CheY_6_ and CheA_3_P1 could be obtained in 100 mM Bicine (pH 9), 1 M LiCl, 20% (w/v) PEG6000. Crystals were optimized using a dilution series of the initial condition. Crystals reached their final size of 400×100×100 µm^3^ after 12 h.

Diffraction data were collected at 100 K, crystals being flash-cooled in a cryo N_2_ gas stream. Before flash-freezing, crystals were cryo-protected with perfluoropolyether oil PFO-X125/03 (Lancaster Synthesis). The native dataset was collected at beamline I03 (λ = 0.9757 Å) and the SeMet dataset at beamline I04 (λ = 0.979 Å) at the Diamond Light Source. Data for the phosphorylated complex were collected on a MAR345 detector (Marresearch) mounted on a Miromax007 generator (Rigaku), equipped with Varimax HR Osmic mirrors (Rigaku). X-ray data were processed and scaled with the HKL suite [46]. Data collection statistics are shown in Table 1.

### Structure Determination and Refinement

The CheA_3_P1-CheY_6_ complex structure was determined by SAD analysis. The positions of 12 selenium atoms were determined by using SHELXD [47]. This solution was put into AUTOSHARP [48] for phase calculation, improvement, and phase extension using the high-resolution native data to 1.4 Å resolution. The resulting map was of high quality and allowed tracing of the whole polypeptide chain. An initial model was built automatically using Arp/wARP [49] and manually adjusted using COOT [50]. The structure was refined using autoBUSTER [51], REFMAC [52], and Phenix [53]. The phosphorylated complex was solved by molecular replacement using Phaser [54] with the unphosphorylated complex as model.

Refinement statistics are given in Table 1; all data within the indicated resolution range were included. Stereochemical properties were assessed by MOLPROBITY [55] and PROCHECK [56]. Ramachandran statistics are as follows (favored/disallowed, %): CheA_3_P1-CheY_6_ unphosphorylated 98.8/0, CheA_3_P1-CheY_6_ phosphorylated 99.1/0. Superpositions were calculated using lsqkab [57] implemented in the CCP4 suite and electrostatic potentials were generated using APBS [58]. Buried surface areas of protein–protein interactions were calculated using the PISA Webserver (http://www.ebi.ac.uk/msd-srv/prot_int/pistart.html). Coordinates are deposited in RSCB Data Bank under 3KYI and 3KYJ.

### Surface Plasmon Resonance Binding Studies

SPR experiments were performed using a Biacore T100 machine (GE Healthcare) at 25°C in standard buffer supplemented with 0.05% (v/v) Tween 20. Protein concentrations were determined from the absorbance at 280 nm using calculated molar extinction coefficients. Proteins for surface attachment were enzymatically biotinylated within an engineered C-terminal tag. These proteins were then attached to surfaces on which 3,000 response units (RU) of streptavidin were coupled via primary amines [59] yielding a density of 200–1,500 RU of biotinylated protein. All experiments were done in duplicates with independently purified proteins. The signal from experimental flow cells was corrected by subtraction of a blank and reference signal from a mock or irrelevant protein-coupled flow cell. In all experiments analyzed, the experimental trace returned to baseline after each injection and the data fitted to a simple 1∶1 Langmuir model of binding. *K*_d_ values were obtained by nonlinear curve fitting of the Langmuir binding isotherm (bound = Cmax/(*K*_d_ + C), where C is analyte concentration and max is the maximum analyte binding) evaluated using the Biacore Evaluation software (GE Healthcare).

### Detection of Phosphotransfer from CheA_3_P1-P to the RRs

Assays were performed at 20°C in TGMNKD buffer (50 mM Tris HCl, 10% [v/v] glycerol, 5 mM MgCl_2_, 150 mM NaCl, 50 mM KCl, 1 mM DTT [pH 8.0]). CheA_3_P1 was phosphorylated using [γ-^32^P] ATP and CheA_4_, and purified as described previously [26]. The final preparation of CheA_3_P1-^32^P was free of ATP and CheA_4_. CheA_3_P1-^32^P (1 µM) was mixed with 5 µM RR in a final reaction volume of 100 µl. Following the addition of RR, reaction aliquots of 10 µl were taken at the indicated time points and quenched immediately in 20 µl of 1.5× SDS-PAGE loading dye (3.75% [w/v] SDS, 45 mM EDTA, 18.75 mM Tris HCl, 18.75% [v/v] glycerol, 1.5% [v/v] β-mercaptoethanol [pH 6.8]). Quenched samples were analyzed using SDS-PAGE and phosphorimaging as described previously [21].

## Supporting Information

Figure S1 **Stereoview of a superposition of the metal binding site of CheY_6_ and** ***E. coli*** **CheY.** CheY_6_ is shown in pale green, *E. coli* CheY with Mg^2+^ bound (PDB-code: 1CHN) in yellow, and *E.coli* CheY without Mg^2+^ (3CHY) in teal. Structures were aligned on their secondary structure elements using secondary structure matching (SSM) implemented in COOT [50]. Residues involved in the coordination of Mg^2+^ in *E. coli* CheY are shown in stick representation for all three structures. Residues in *E. coli* structures are only labelled for the Mg^2+^ bound form. Mg^2+^ from the *E. coli* structure is shown as a grey sphere. CheY_6_ resembles the Mg^2+^ bound form of *E. coli* CheY despite not having a divalent cation bound to its metal binding site.
(6.24 MB TIF)

Figure S2 **Electron density of phosphorylated His51 in CheA_3_P1 in the active complex structure.** The orientation is similar to Figure 2B. The density represents a 2*Fobs-Fcalc* map contoured at 1.5 σ and calculated after initial rigid body refinement in Phaser [54] and one round of positional refinement in autoBUSTER [51], both omitting the phosphate group from the model.
(0.64 MB TIF)

Figure S3 **Binding of CheA_3_P1 to substitution mutants of CheY_4_ and CheY_6_.** (A–C) Binding of CheA_3_P1 to CheY_4_(P12A,S13M,C16L,M17Y), CheY_6_(M13S), and CheY_6_(M13S,L16S,Y17M). Left, representative sets of experimental sensorgrams from typical equilibrium-based binding experiments, with reference subtraction. Different concentrations of CheA_3_P1 were injected over surfaces coupled with the respective RR. For all injections, the experimental traces reached equilibrium and returned to baseline after the injection. Right, plot of the equilibrium binding response (response units [RU]) against CheA_3_P1 concentration ranging from 120 nM to 2 mM. Within one experiment, each concentration was measured twice. All experiments were performed in duplicate. Best-fit binding curves corresponding with a 1∶1 binding model are shown as lines. Experiments with CheY_6_(M13S) and CheY_6_(M13S,L16S,Y17M) did not reach saturation due to very low affinity, thus the *K_d_* value is estimated.
(0.52 MB TIF)

Figure S4 **Superposition of the CheA_3_P1-CheY_6_ complex structure with the Spo0B-Spo0F complex and YPD1-SLN1 complex.** (A) Superposition of CheA_3_P1-CheY_6_ with Spo0B-Spo0F (PDB-code: 1F51). (B) Superposition of CheA_3_P1-CheY_6_ with YPD1-SLN1 (1OXB). All structures were superimposed on the RRs using SSM as implemented in COOT [50]. CheY_6_ is shown in pale green, CheA3P1 in light blue, Spo0F in orange, Spo0B in teal, SLN1 in purple, and YPD1 in aquamarine. Only the four-helix bundle of the Spo0B dimer is shown; the C-terminal domain is omitted for clarity. The orientation is similar to Figure 1. CheA_3_P1 is structurally more similar to the monomeric YPD1 than to Spo0B.
(2.64 MB TIF)

## Abbreviations

RR: response regulator
SPR: surface plasmon resonance
