# Supplementary figures and images for: Using Structural Information to Change the Phosphotransfer Specificity of a Two-Component Chemotaxis Signalling Complex
Source: PLoS Biol. 2010 Feb 9;8(2):e1000306. doi: 10.1371/journal.pbio.1000306 (PMC2817712; doi:10.1371/journal.pbio.1000306)

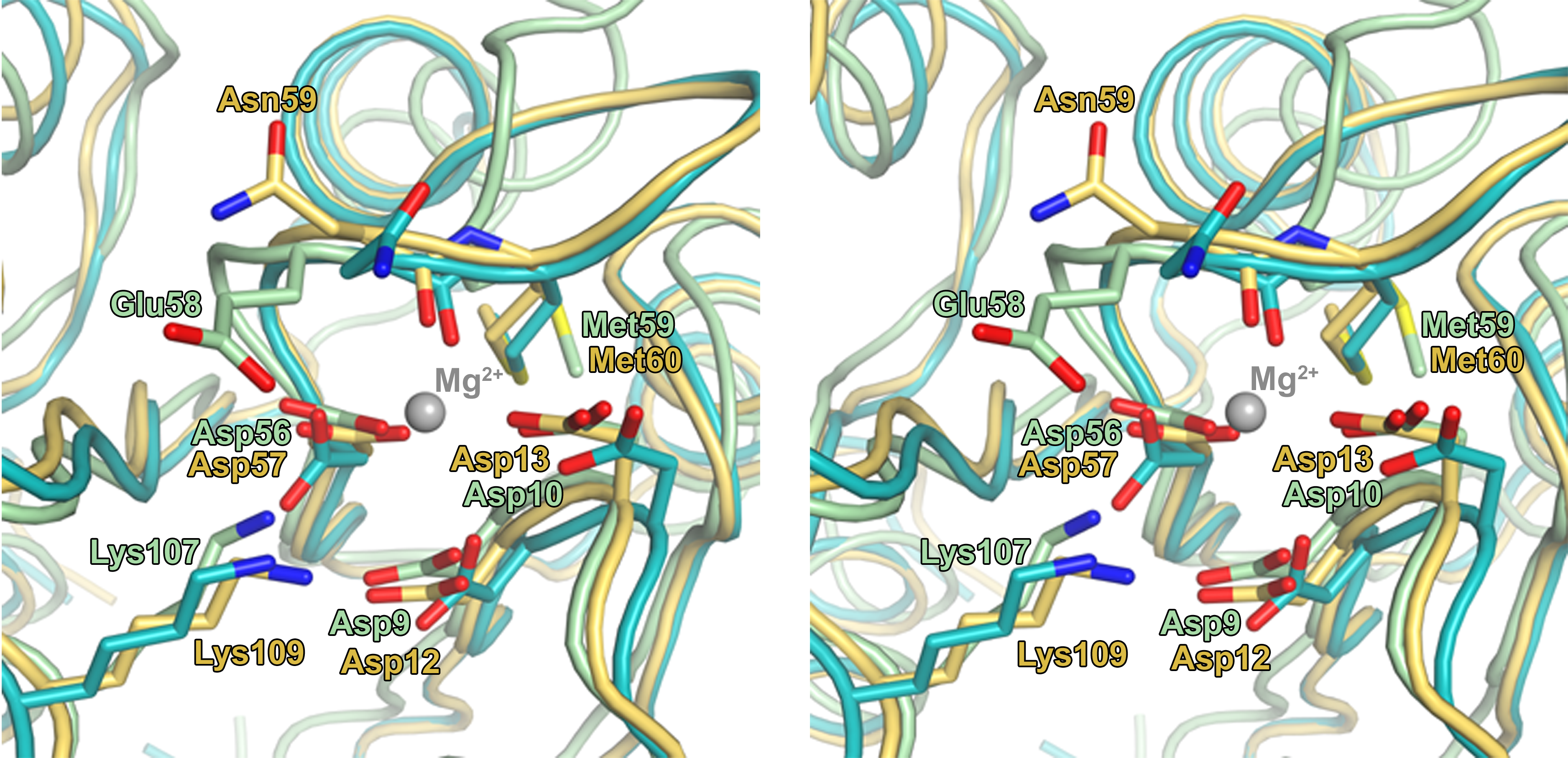

Supplement: Figure S1 — Stereoview of a superposition of the metal binding site of CheY6 and E. coli CheY. CheY6 is shown in pale green, E. coli CheY with Mg2+ bound (PDB-code: 1CHN) in yellow, and E.coli CheY without Mg2+ (3CHY) in teal. Structures were aligned on their secondary structure elements using secondary structure matching (SSM) implemented in COOT [50]. Residues involved in the coordination of Mg2+ in E. coli CheY are shown in stick representation for all three structures. Residues in E. coli structures are only labelled for the Mg2+ bound form. Mg2+ from the E. coli structure is shown as a grey sphere. CheY6 resembles the Mg2+ bound form of E. coli CheY despite not having a divalent cation bound to its metal binding site. (6.24 MB TIF) [file pbio.1000306.s001.tif]

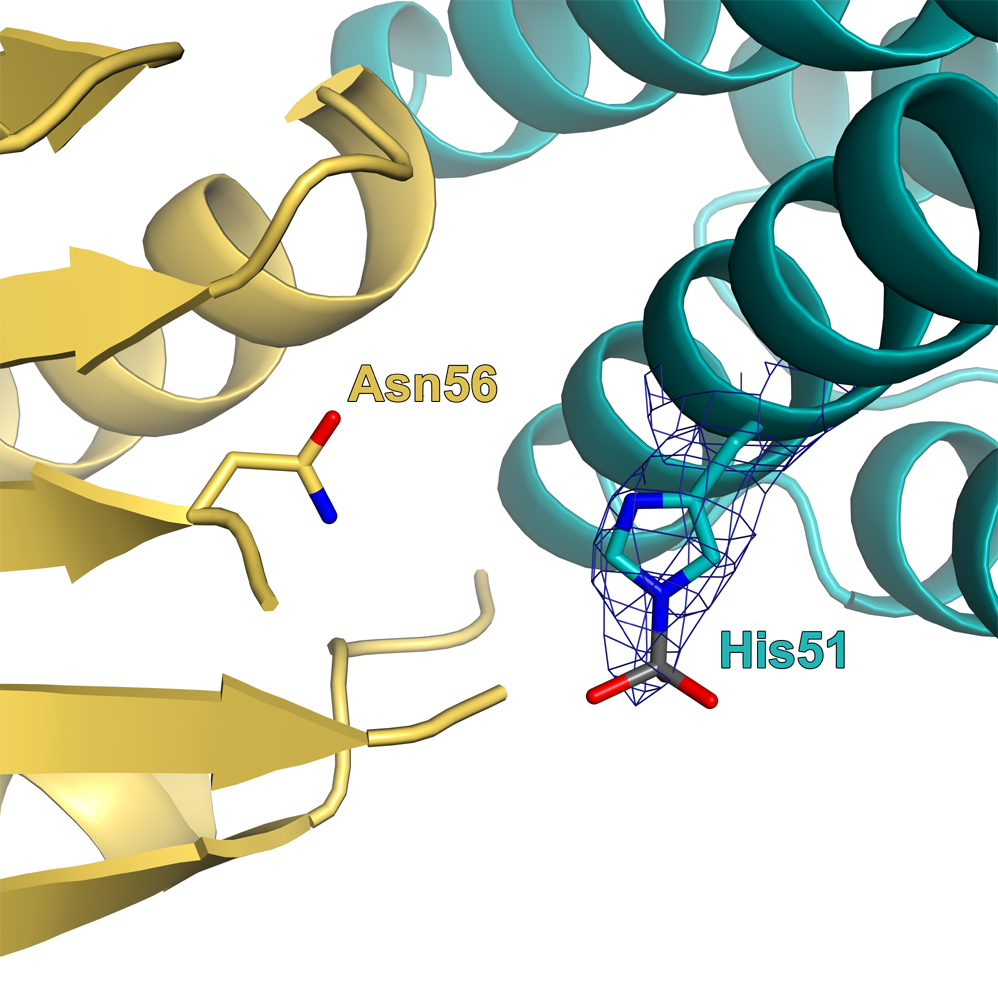

Supplement: Figure S2 — Electron density of phosphorylated His51 in CheA3P1 in the active complex structure. The orientation is similar to Figure 2B. The density represents a 2Fobs-Fcalc map contoured at 1.5 σ and calculated after initial rigid body refinement in Phaser [54] and one round of positional refinement in autoBUSTER [51], both omitting the phosphate group from the model. (0.64 MB TIF) [file pbio.1000306.s002.tif]

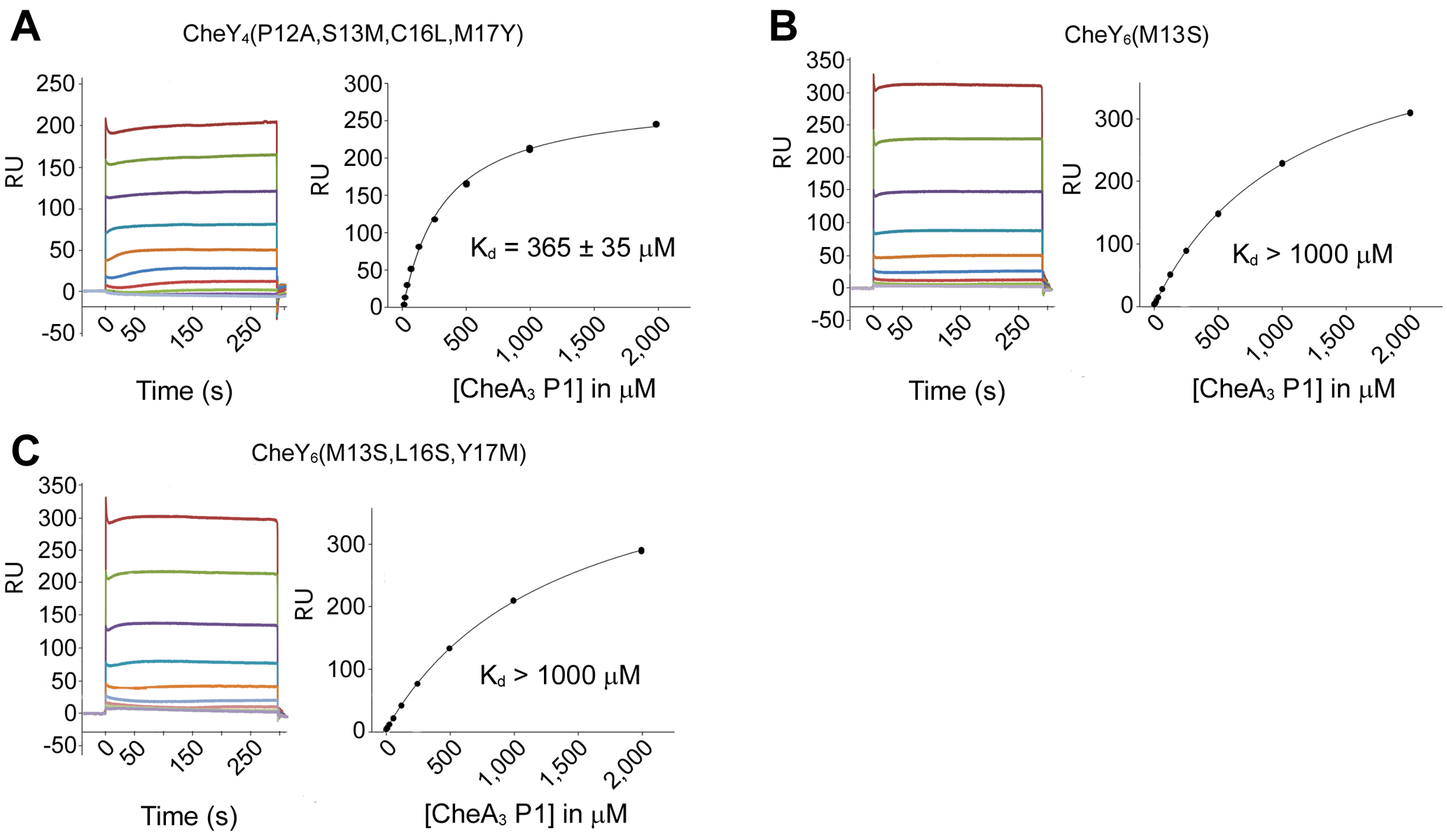

Supplement: Figure S3 — Binding of CheA3P1 to substitution mutants of CheY4 and CheY6. (A–C) Binding of CheA3P1 to CheY4(P12A,S13M,C16L,M17Y), CheY6(M13S), and CheY6(M13S,L16S,Y17M). Left, representative sets of experimental sensorgrams from typical equilibrium-based binding experiments, with reference subtraction. Different concentrations of CheA3P1 were injected over surfaces coupled with the respective RR. For all injections, the experimental traces reached equilibrium and returned to baseline after the injection. Right, plot of the equilibrium binding response (response units [RU]) against CheA3P1 concentration ranging from 120 nM to 2 mM. Within one experiment, each concentration was measured twice. All experiments were performed in duplicate. Best-fit binding curves corresponding with a 1∶1 binding model are shown as lines. Experiments with CheY6(M13S) and CheY6(M13S,L16S,Y17M) did not reach saturation due to very low affinity, thus the Kd value is estimated. (0.52 MB TIF) [file pbio.1000306.s003.tif]

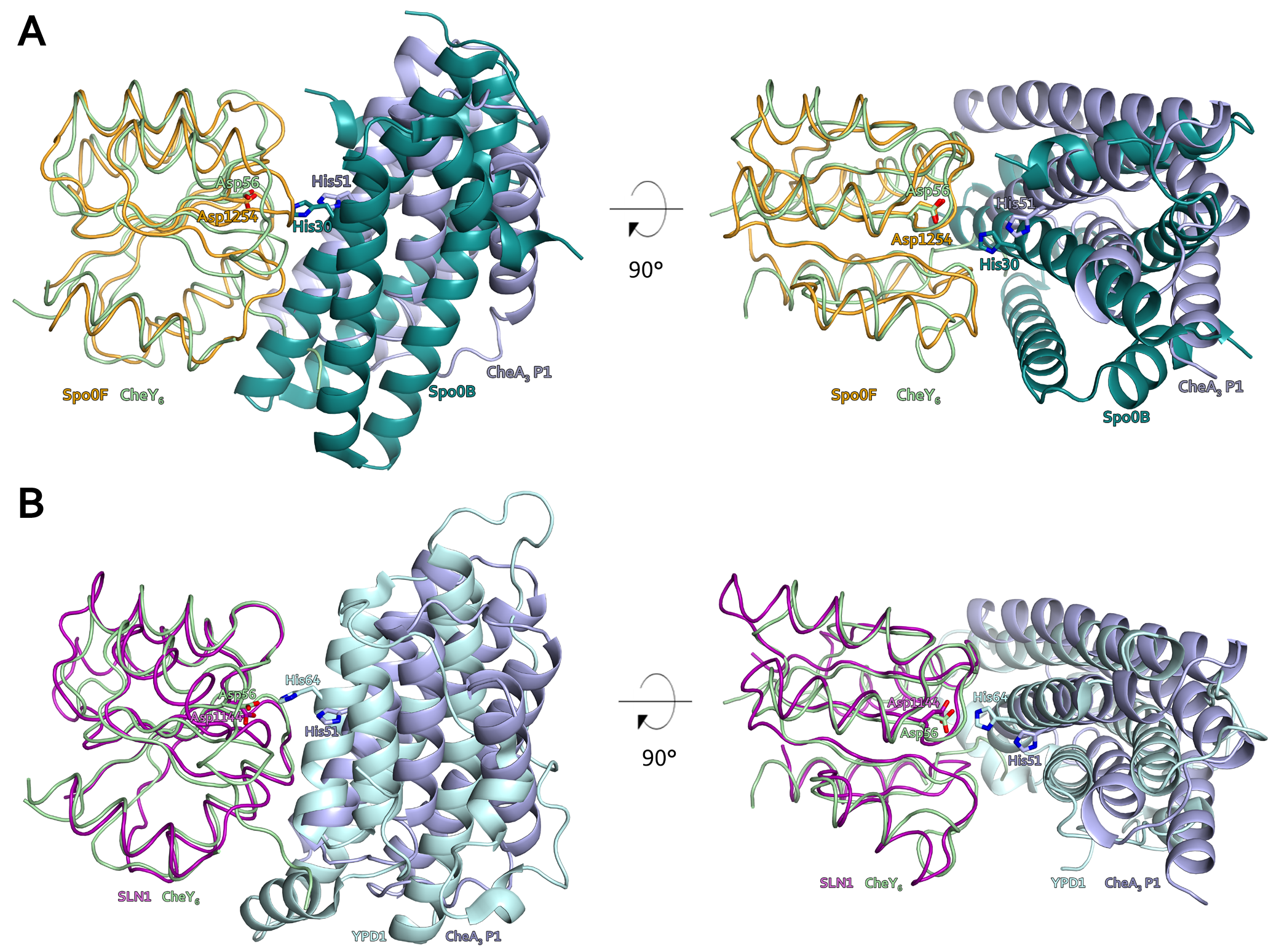

Supplement: Figure S4 — Superposition of the CheA3P1-CheY6 complex structure with the Spo0B-Spo0F complex and YPD1-SLN1 complex. (A) Superposition of CheA3P1-CheY6 with Spo0B-Spo0F (PDB-code: 1F51). (B) Superposition of CheA3P1-CheY6 with YPD1-SLN1 (1OXB). All structures were superimposed on the RRs using SSM as implemented in COOT [50]. CheY6 is shown in pale green, CheA3P1 in light blue, Spo0F in orange, Spo0B in teal, SLN1 in purple, and YPD1 in aquamarine. Only the four-helix bundle of the Spo0B dimer is shown; the C-terminal domain is omitted for clarity. The orientation is similar to Figure 1. CheA3P1 is structurally more similar to the monomeric YPD1 than to Spo0B. (2.64 MB TIF) [file pbio.1000306.s004.tif]
